# Complex infective endocarditis with perivalvular abscess and fistulation: two high-risk cases requiring extensive reconstruction and valve replacement

**DOI:** 10.1186/s13019-026-04491-1

**Published:** 2026-07-19

**Authors:** Marco Lizwan, Edwin Yosef Widjaja, See Hooi Ewe, Thuan Tong Tan, Teing Ee Tan, Philip Y. K. Pang

**Affiliations:** 1 Department of Cardiothoracic Surgery, National Heart Centre Singapore, 5 Hospital Dr, Singapore, 169609 Singapore; 2 Department of Cardiothoracic Surgery and Vascular Surgery, Faculty of Medicine, Dr. Soetomo General Academic Hospital, Airlangga University, Surabaya, Indonesia; 3 Department of Cardiology, National Heart Centre Singapore, 169609 Singapore, Singapore; 4 Department of Infectious Disease, Singapore General Hospital, 169608 Singapore, Singapore

**Keywords:** Infective endocarditis, Perivalvular abscess, LVOT-right atrial fistula, Aortomitral curtain reconstruction, Multivalve surgery

## Abstract

**Background:**

Infective endocarditis (IE) complicated by aortic root abscess, fistulation and multivalve involvement is rare but carries high mortality. Fistulation from the left ventricular outflow tract (LVOT) to the right atrium (RA) represents an exceptionally uncommon sequela due to extensive perivalvular tissue destruction. Early surgical intervention remains crucial, but postoperative outcomes vary widely depending on host factors, organism control and the feasibility of durable reconstruction.

**Case presentation:**

We report two cases of severe aortic valve endocarditis with aortic root abscess and acquired LVOT-RA fistula. The first patient had native-valve *Streptococcus mitis* endocarditis and underwent aortic and tricuspid valve replacement with double CardioCel patch repair of the fistula. He recovered after transient renal and hepatic dysfunction and completed six weeks of intravenous ceftriaxone. The second patient had prosthetic aortic valve endocarditis with aortomitral curtain destruction, pseudoaneurysm, severe malnutrition, and tricuspid involvement. He underwent redo sternotomy, aortomitral curtain reconstruction, double patch fistula repair, and triple bioprosthetic valve replacement. Although the initial repair was technically satisfactory, he later developed severe mitral paravalvular regurgitation with recurrent *Staphylococcus haemolyticus* bacteremia and died after deterioration with sepsis and multiorgan dysfunction.

**Conclusion:**

These cases illustrate the range of outcomes after complex endocarditis surgery. Patch-based reconstruction may be appropriate when radical debridement leaves sufficient tissue for secure prosthetic seating, whereas extensive prosthetic infection, malnutrition, recurrent bacteremia, and tissue friability increase the risk of late mechanical failure.

**Supplementary Information:**

The online version contains supplementary material available at https://doi.org/10.1186/s13019-026-04491-1.

## Introduction

Perivalvular extension of infective endocarditis (IE) – including annular or aortic root abscess, pseudoaneurysm and intracardiac fistula – worsens prognosis and frequently mandates surgical intervention. Contemporary guidelines recommend early surgery when there is heart failure, uncontrolled infection or perivalvular complications such as abscess or fistulation [1, 2].

The operative strategy varies from targeted debridement with patch reconstruction to aortic root replacement or aortomitral curtain reconstruction, depending on the extent of infection and the quality of residual tissue [3–5]. These options are not interchangeable: patch repair may shorten an already hazardous operation and preserve coronary anatomy, whereas root replacement or hemi-Commando/Commando reconstruction may be required when annular or intervalvular destruction prevents secure valve implantation [4, 5].

We present two contemporaneous cases of severe multivalvular IE complicated by aortic root abscess and left ventricular outflow tract – right atrial (LVOT-RA) fistulation requiring extensive reconstruction. The paired format is intended to be illustrative rather than comparative. It allows discussion of surgical judgment, reconstructive options, infectious disease considerations, and patient factors that may influence outcomes in similarly destructive disease.

## Case presentation

Written informed consent was obtained from patient to include the information in this manuscript. Institutional Review Board (IRB) approval was not required.

### Case 1

**Fig. 1:**
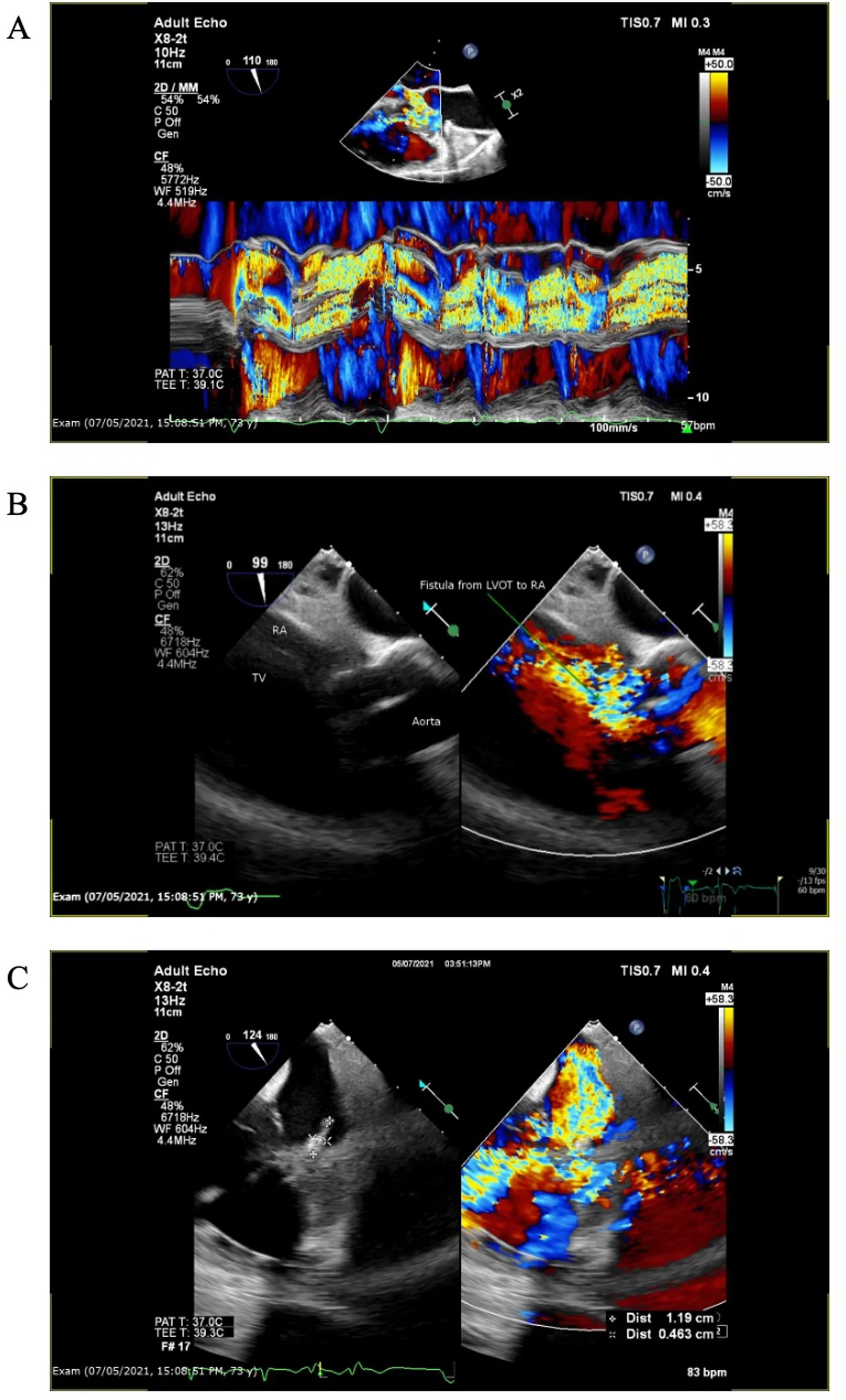
(**A**) Pre-operative transesophageal echocardiography (TEE) demonstrating a large mobile vegetation on the right coronary cusp of the aortic valve, associated with severe aortic regurgitation. (**B**) TEE showing fistulous communication between the left ventricular outflow tract and the right atrium, with color Doppler demonstrating continuous left-to-right shunting. (**C**) TEE demonstrating vegetation attached to the septal leaflet of the tricuspid valve, with associated leaflet destruction contributing to significant tricuspid regurgitation

A 73-year-old man with chronic aortic regurgitation presented with one-month history of progressive dyspnea, fever, leg swelling and cardiogenic shock. He was of average body habitus (body mass index (BMI) 23.7 kg/m^2^) but appeared clinically frail. Blood cultures grew *Streptococcus mitis*. Echocardiography demonstrated severe aortic regurgitation with large vegetations on the right coronary cusp, a periaortic abscess (Fig. 1A, Video 1), and a LVOT-RA fistulation (Fig. 1B, Video 1), and additional tricuspid valve vegetations with moderate-severe regurgitation (Fig. 1C, Video 1). He developed acute kidney injury requiring continuous renal replacement therapy, metabolic acidosis, and hepatic dysfunction. After empirical benzylpenicillin, cloxacillin, and gentamicin, antimicrobial therapy was narrowed to ceftriaxone based on susceptibility results.

**Fig. 2:**
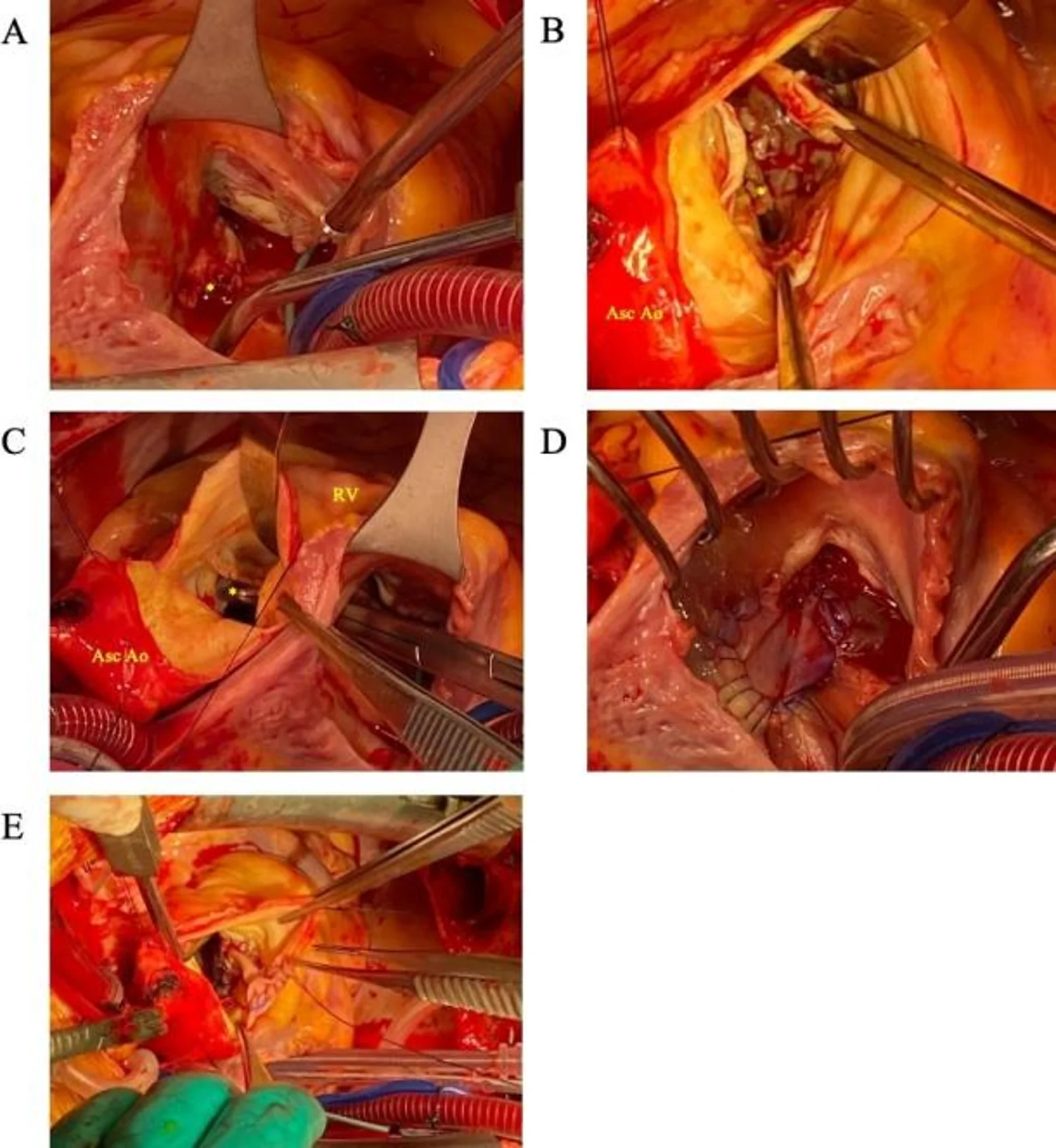
(**A-B**) Extensive infective endocarditis of the aortic valve with a 2 cm perforation of the right coronary cusp and destruction of its free edge (surgeon’s view). (**C**) Tip of the right-angled forceps showing the fistulous communication from left ventricular outflow tract at the vicinity of the membranous septum and penetrating bundle communicating freely with the right atrium at the level of tricuspid annulus, adjacent to the septal tricuspid leaflet. (**D-E**) CardioCel patch repair of the left ventricular outflow tract – right atrial fistulation

Urgent surgery was performed after hemodynamic stabilization. Intraoperative findings confirmed extensive destruction of the right coronary cusp and annulus, a 2 × 2 cm perforated abscess at the membranous septal region, and free communication into the right atrium at the level of the tricuspid annulus (Fig. 2A-C). Aortic root replacement was not performed because the infection was not circumferential, the ascending aorta was soft, and both coronary ostia were well visualized with sufficient residual root tissue for secure bioprosthetic implantation after debridement. Radical debridement was followed by double CardioCel patch repair of the LVOT-RA fistula (Fig. 2D-E). The aortic valve was replaced with a 23-mm Perimount Magna Ease bioprosthesis and the tricuspid valve with a 31-mm Perimount Magna Ease bioprosthesis. Intraoperative transesophageal echocardiogram (TEE) showed satisfactory prosthetic function, intact patch repairs, and no residual shunt.

Postoperatively, organ dysfunction resolved, and he was extubated on postoperative day 2. He had complete heart block which necessitated implantation of a cardiac resynchronization therapy pacemaker. Follow-up transthoracic echocardiography (TTE) showed satisfactory aortic and tricuspid prosthetic gradients, trivial prosthetic regurgitation, no ventricular septal defect, and no residual fistula. He completed six weeks of ceftriaxone from operative source control and was discharged to outpatient parenteral antibiotic therapy and rehabilitation. Follow-up blood cultures remained negative.

### Case 2

A 66-year-old man with prior 23-mm bioprosthetic aortic valve replacement presented with fever, dyspnea, and lethargy. His medical history included chronic hepatitis C infection and prior intravenous drug use. He was markedly cachectic, with a body mass index of 15.4 kg/m², minimal subcutaneous fat noted intraoperatively, and preoperative hypoalbuminemia (albumin 27 g/L). Blood cultures grew *Streptococcus mitis* and cleared after ampicillin and gentamicin. Echocardiography demonstrated prosthetic aortic valve endocarditis with multiple vegetations, extensive aortic root abscess involving the aortomitral curtain, a 2.2-cm posterior aortic root pseudoaneurysm, an acquired LVOT-RA fistula, and tricuspid valve involvement. Computed tomography identified a splenic infarct. Coronary angiography showed borderline coronary artery disease and an anomalous right coronary artery arising from the left coronary cusp with an interarterial course.

**Fig. 3:**
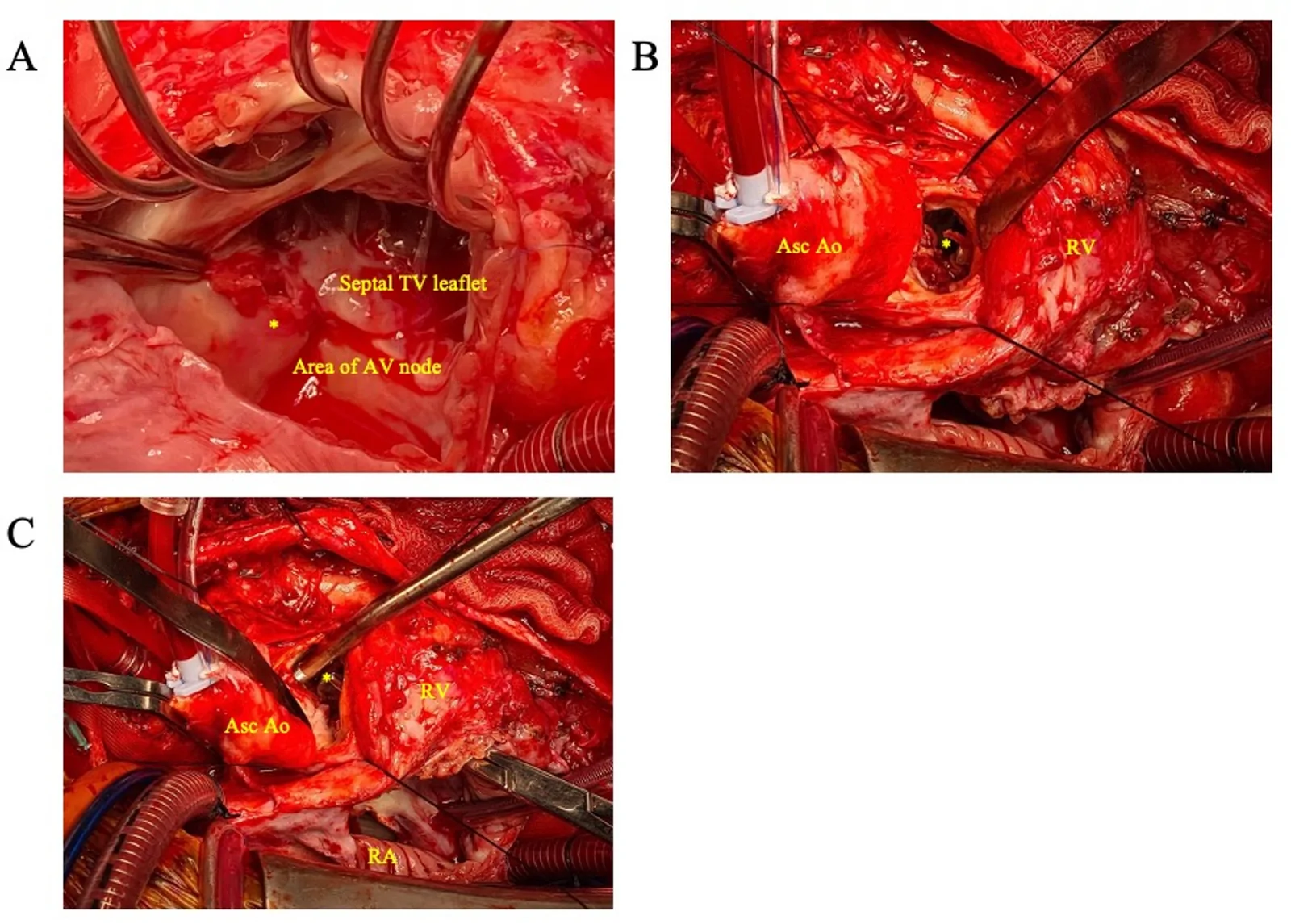
(**A**) Vegetations at the 7 o’clock position of the septal tricuspid leaflet (surgeon’s view), surrounding the opening of the fistula from the aortic root to the right atrium. (**B**) Extensive vegetations encasing the aortic annulus from the 3 to 11 o’clock position (surgeon’s view). (**C**) Tip of the right-angled forceps showing the fistulous communication between the right atrium and aortic root

He underwent high-risk redo sternotomy after multidisciplinary and family discussions. Operative findings included dense adhesions, severe tissue friability, prosthetic aortic valve endocarditis with vegetations on both aortic and ventricular surfaces, a periaortic abscess extending into the aortomitral curtain, and complete detachment of the anterior mitral leaflet after debridement of unhealthy tissue (Fig. 3). A 1.5 × 1.5 cm perforated abscess created a fistula from the LVOT near the membranous septum and penetrating bundle to the right atrium at the tricuspid annulus. A separate tricuspid vegetation involved the septal leaflet adjacent to the atrioventricular node, precluding repair.

The reconstructive strategy was patch-based rather than root replacement. Root replacement would have required extensive coronary mobilization and reimplantation in a redo field with anomalous coronary anatomy, profound malnutrition, active prosthetic infection, and already prolonged operative risk. After debridement, the remaining root and annular tissue were judged sufficient for valve implantation and intervalvular fibrosa reconstruction. A dedicated CardioCel patch was used to reconstruct the aortomitral curtain and support the anterior mitral prosthetic annulus. The LVOT-RA tract was then closed with a double CardioCel patch technique from the left-sided outflow tract and right atrial aspects. The aortic, mitral, and tricuspid valves were replaced with 19-mm, 27-mm, and 29-mm Perimount Magna Ease bioprostheses, respectively. Post-replacement TEE demonstrated satisfactory aortic, mitral, and tricuspid prosthetic function, no paravalvular leak, intact aortic and right atrial patch repairs, intact aortomitral continuity, and no residual shunt. Cross-clamp and cardiopulmonary bypass times were 355 and 482 min, respectively.

The early postoperative course was complicated by vasoplegia, acute kidney injury requiring renal replacement therapy, ischemic hepatitis, atrial flutter with right atrial thrombus, gastrointestinal bleeding, and complete heart block requiring left bundle branch area pacemaker implantation. He completed six weeks of ceftriaxone, followed by oral amoxicillin-clavulanate until discharge. Renal function improved sufficiently for dialysis catheter removal, and he was discharged to a community hospital for rehabilitation.

Two months later, he was readmitted with dyspnea, hypoxemia, pulmonary edema, and acute-on-chronic kidney injury. TEE demonstrated a 10 × 8 mm defect at the reconstructed aortomitral curtain adjacent to the mitral prosthesis, with severe anterior mitral paravalvular regurgitation, systolic pulmonary vein flow reversal, and preserved transmitral gradient (Video 3). Positron emission tomography-computed tomography showed suboptimal myocardial suppression and no definite cardiac focus of active infection within the limitations of the study. The clinical trajectory then changed with high-grade *Staphylococcus haemolyticus* bacteremia from central and peripheral cultures. This coagulase-negative Staphylococcus was distinct from the initial *Streptococcus mitis* infection and was considered more consistent with healthcare-associated bacteremia with possible prosthetic involvement than microbiological relapse. Antibiotics were escalated from piperacillin-tazobactam to meropenem and vancomycin. Although percutaneous closure was initially considered, progressive sepsis, thrombocytopenia, recurrent dialysis-requiring renal failure, respiratory failure, and uncertain infection control made further intervention prohibitive. After multidisciplinary and family discussions, care was redirected to comfort-focused management, and he died during the admission.

## Discussion

These cases demonstrate the heterogeneity of complex endocarditis surgery. They should not be interpreted as evidence that one reconstructive method is superior to another. Rather, they illustrate how the same broad anatomical problem – aortic root abscess with LVOT-RA fistula – may require different operative judgments depending on native versus prosthetic infection, residual tissue quality, coronary anatomy, nutritional reserve, organism control, and postoperative complications. Current guidelines support early surgical evaluation for IE complicated by heart failure, abscess, pseudoaneurysm, or fistula, but the choice between patch reconstruction and root replacement remains individualized [1, 2].

Intervalvular fibrosa (aortomitral curtain) reconstruction using biological patches (including bovine pericardium and decellularized xenografts such as CardioCel) has been reported with acceptable mid-term durability in selected patients [4]. Nevertheless, patch-based repair in active infection carries a risk of recurrent paravalvular leak or prosthesis dehiscence, particularly when tissue planes are friable or infection recurs. In extensive circumferential disease, some advocate root replacement to reduce recurrence risk, though at the expense of greater operative complexity [5].

In Case 1, patch repair was selected because the destructive process was localized to the right coronary cusp, adjacent annulus, and membranous septal region. The coronary ostia were preserved, the ascending aorta was soft, and the remaining aortic root allowed stable valve seating. In this setting, root replacement would have increased operative complexity without clear anatomical necessity. In Case 2, the decision was more finely balanced. A hemi-Commando or Commando-type reconstruction is an established option when invasive double-valve endocarditis destroys the aortomitral curtain, particularly when secure mitral or aortic prosthetic anchoring cannot otherwise be achieved [4]. However, the patient had a redo sternotomy, profound cachexia, hypoalbuminemia, active prosthetic infection, anomalous coronary anatomy, and extremely friable tissue. The operative strategy therefore prioritized radical debridement, restoration of aortomitral continuity with CardioCel, and avoidance of additional coronary button manipulation.

LVOT-RA fistulation is an uncommon but severe complication of aortic valve endocarditis [6]. Such lesions are anatomically related to acquired Gerbode-type defects because they often traverse or abut the membranous septum; however, the tract in endocarditis may extend through aortic root or periannular abscess cavities rather than representing a simple congenital left ventricular-right atrial defect [6]. This distinction is clinically relevant because treatment requires debridement of infected tissue, closure of the abscess-associated tract, and secure prosthetic valve implantation rather than isolated septal defect closure. The close relationship to the conduction tissue also explains the high risk of postoperative heart block in both cases [3, 6].

Our second case also highlights the limitation of imaging in excluding persistent or recurrent infection. The positron emission tomography-computed tomography study did not demonstrate a definite cardiac focus, but myocardial suppression was suboptimal. This result lowered suspicion for uncontrolled *Streptococcus mitis* relapse but did not exclude prosthetic infection, especially once high-grade *Staphylococcus haemolyticus* bacteremia developed. Coagulase-negative staphylococci are typical pathogens in prosthetic valve endocarditis and may reflect healthcare-associated acquisition, line-related bacteremia, or prosthetic colonization [7, 8]. In the context of a newly reconstructed aortomitral curtain and severe paravalvular regurgitation, recurrent bacteremia substantially worsened the risk-benefit profile of both redo surgery and percutaneous closure.

The contrasting outcomes likely reflect a combination of surgical and host factors. The first patient had native-valve IE, localized root destruction, microbiological control, and recovery of renal and hepatic function. The second patient had prosthetic valve IE, redo surgery, extensive aortomitral curtain destruction, a markedly low body mass index, hypoalbuminemia, and recurrent bacteremia with a new organism. Severe malnutrition and hypoalbuminemia plausibly contributed to impaired tissue healing and vulnerability to dehiscence, but causality cannot be established from two cases. This distinction has been made explicit because the present report is hypothesis-generating and should be interpreted alongside larger surgical series and guideline-based evidence [1, 3–5].

## Conclusion

Multivalve IE with aortic root abscess and LVOT-RA fistula represents a surgical emergency with heterogenous outcomes. Aggressive debridement and patch-based reconstruction can be durable when residual tissue is suitable and infection is controlled. In prosthetic infection with aortomitral curtain destruction, malnutrition, recurrent bacteremia, and tissue friability, even technically successful reconstruction may fail. Surgical strategy should be individualized, with explicit consideration of patch repair, root replacement, hemi-Commando or Commando reconstruction, and the patient’s physiological reserve.

## Supplementary Information

Below is the link to the electronic supplementary material.

Supplementary Material 1: Video 1. Pre-operative transesophageal echocardiography (TEE) demonstrating large mobile vegetation on the right coronary cusp of the aortic valve, associated with severe aortic regurgitation. There was also extensive infective involvement of the left ventricular outflow tract with a periaortic root abscess adjacent to the aortic annulus. Color and continuous-wave doppler TEE demonstrated continuous flow between the left ventricular outflow tract and the right atrium (LVOT-RA) fistula.

Supplementary Material 2: Video 2. Postoperative transthoracic echocardiography (TTE) following aortic and tricuspid valve replacement with with double CardioCel patch repair of the LVOT-RA fistula. Color Doppler imaging demonstrates a well-seated, normally functioning aortic prosthesis with no paravalvular leak. There is no residual communication between the left ventricular outflow tract and the right atrium, confirming successful fistula closure. The tricuspid prosthesis is well aligned with satisfactory leaflet motion and no evidence of residual regurgitation or shunt.

Supplementary Material 3: Video 3. TEE demonstrating dehiscence of the mitral valve prosthesis.

## Data Availability

No datasets were generated or analysed during the current study.

## Abbreviations

BMI: Body mass index
CT: Computed tomography
IE: Infective endocarditis
LVOT-RA: Left ventricular outflow tract – right atrium
TEE: Transesophageal echocardiography
TTE: Transthoracic echocardiography
